# Supplementary material for: Responsiveness to endurance training can be partly explained by the number of favorable single nucleotide polymorphisms an individual possesses
Source: PLoS One. 2023 Jul 20;18(7):e0288996. doi: 10.1371/journal.pone.0288996 (PMC10358902; doi:10.1371/journal.pone.0288996)
Supplement: S1 File — Pre COVID-19 application and approval. (PDF) [file pone.0288996.s001.pdf]

Henry Chung
Flat 8, The Forum
Tiverton Way
Cambridge
Cambridgeshire
CB1 3HT

Anglia Ruskin University
ARU Chelmsford
Bishop Hall Lane
CM1 1SQ
www.aru.ac.uk

16th December 2019

Dear Henry,

Principal Investigator: Henry Chung
FREP/SREP number: FSE/FREP/19/864
Project Title: Physiological and metabolic responses to exercise programs based on genotype

Thank you for supplying revisions to your application for ethical approval, as requested by the Faculty Research Ethics Panel (FREP) following its meeting on 22/10/2019.

I am pleased to inform you that your ethics application has been approved by the Faculty Research Ethics Panel (FREP) under the terms of Anglia Ruskin University's Research Ethics Policy (dated 1 May 2019, Version 1.10).

Ethical approval is given for a period of 3 years for doctorate students from 16/12/2019. If your research will extend beyond this period, it is your responsibility to apply for an extension before your approval expires.

It is your responsibility to ensure that you comply with Anglia Ruskin University's Research Ethics Policy and the Code of Practice for Applying for Ethical Approval at Anglia Ruskin University available at www.anglia.ac.uk/researchethics including the following:

- The procedure for submitting substantial amendments to the committee, should there be any changes to your research. You cannot implement these amendments until you have received approval from the FREP for them.
- The procedure for reporting accidents, adverse events and incidents.
- The General Data Protection Requirement (GDPR) if your research will take place in the European Economic Area (EEA)[1] or involve sending or bringing any personal data[2] into it. If your research will take place in the UK or involve sending or

[1] The EEA includes EU member states and also Iceland, Liechtenstein and Norway.
[2] Personal data means any information relating to an identified or identifiable natural person ('data subject'); an identifiable natural person is one who can be identified, directly or indirectly, in particular by reference to an identifier such as a name, an identification number, location data, an online

a.r.u. | Anglia Ruskin University

ARU Chelmsford
Bishop Hall Lane
CM1 1SQ
www.aru.ac.uk

bringing any personal data into it, you must also comply with the Data Protection Act (2018). Other countries in the EEA may have further data protection legislation you must comply with. If your research will take place outside the EEA, you must comply with any data protection legislation relating to that country or countries.

- Any other legislation relevant to your research. You must also ensure that you are aware of any emerging legislation relating to your research and make any changes to your study (which you will need to obtain ethical approval for) to comply with this.
- Obtaining any further ethical approval required from the organisation or country (if not carrying out research in the UK) where you will be carrying the research out. This includes other Higher Education Institutions if you intend to carry out any research involving their students, staff or premises. Please ensure that you send the FREP copies of this documentation if required, prior to starting your research.
- Any laws of the country where you are carrying the research and obtaining any other approvals or permissions that are required.
- Any professional codes of conduct relating to research or requirements from your funding body (please note that for externally funded research, where the funding has been obtained via Anglia Ruskin University, a Project Risk Assessment must have been carried out prior to starting the research).
- Completing a Risk Assessment (Health and Safety) if required and updating this annually or if any aspects of your study change which affect this.
- Notifying the FREP Secretary when your study has ended.

Please also note that your research may be subject to monitoring.

Should you have any queries, please do not hesitate to contact me. May I wish you the best of luck with your research.

Yours sincerely,

Prof Peter Bright
Faculty of Science and Engineering FREP Chair

Email: Peter.Bright@anglia.ac.uk Tel: 01223 698647

Administrative queries should be sent to: SE-Ethics@anglia.ac.uk

---

identifier or to one or more factors specific to the physical, physiological, genetic, mental, economic, cultural or social identity of that natural person.
