# Supplementary material for: Responsiveness to endurance training can be partly explained by the number of favorable single nucleotide polymorphisms an individual possesses
Source: PLoS One. 2023 Jul 20;18(7):e0288996. doi: 10.1371/journal.pone.0288996 (PMC10358902; doi:10.1371/journal.pone.0288996)
Supplement: S2 File — The protocol to complete the project was edited in response to COVID-19. (PDF) [file pone.0288996.s002.pdf]

Principal investigator:	Henry Chung
Project supervisor:	Dr Dan Gordon
Project title:	Physiological responses to exercise programs based on genotype.
SREP code:	ESPGR-19
Approval date	18-12-2020

Application decision: **Approve** under the terms of Anglia Ruskin University's Research Ethics Policy (Dated 8 September 2016, Version 1.7). Approval by SREP is subject to ratification by the FREP.

All documents (PIS, Consent form, Debrief) given to participants, must have an Anglia Ruskin University header.

Any advert must contain the following statement:
The study has received ethics approval by the School Research Ethics Panel (SREP) and ratified by the Faculty Research Ethics Panel under the terms of Anglia Ruskin University's Policy and Code of Practice for the Conduct of Research with Human Participants

If you make changes to any aspect of your approved research, it is important that you discuss this with your supervisor as they can advise you on whether you need any additional ethical approval.

Ethical approval is given for a period of 3 years for postgraduate students. If your research will extend beyond this period, it is your responsibility to apply for an extension before your approval expires.

It is your responsibility to ensure that you comply with Anglia Ruskin University's Research Ethics Policy and the Code of Practice for Applying for Ethical Approval at Anglia Ruskin University available at www.anglia.ac.uk/researchethics including the following.

- The procedure for submitting substantial amendments to the committee, should there be any changes to your research. You cannot implement these amendments until you have received approval from SREP for them.
- The procedure for reporting accidents, adverse events and incidents.
- The General Data Protection Requirement and Data Protection Act (2018).
- Any other legislation relevant to your research. You must also ensure that you are aware of any emerging legislation relating to your research and make any changes to your study (which you will need to obtain ethical approval for) to comply with this.
- Obtaining any further ethical approval required from the organisation or country (if not carrying out research in the UK) where you will be carrying the research out. This includes other Higher Education Institutions if you intend to carry out any research

involving their students, staff or premises. Please ensure that you send the FREP/SREP copies of this documentation if required, prior to starting your research.

- Any laws of the country where you are carrying the research and obtaining any other approvals or permissions that are required.
- Any professional codes of conduct relating to research or requirements from your funding body (please note that for externally funded research, where the funding has been obtained via Anglia Ruskin University, a Project Risk Assessment must have been carried out prior to starting the research).
- Completing a Risk Assessment (Health and Safety) if required and updating this annually or if any aspects of your study change which affect this.
- Notifying the SREP Secretary when your study has ended.

Please also note that your research may be subject to monitoring.

Should you have any queries, please do not hesitate to contact me. May I wish you the best of luck with your research.

Yours sincerely,

SREP Chair
