# Supplementary material for: Responsiveness to endurance training can be partly explained by the number of favorable single nucleotide polymorphisms an individual possesses
Source: PLoS One. 2023 Jul 20;18(7):e0288996. doi: 10.1371/journal.pone.0288996 (PMC10358902; doi:10.1371/journal.pone.0288996)
Supplement: S1 Data — Individual data collected for each participant. (PDF) [file pone.0288996.s003.pdf]

S3. Participant data. Individual data collected for each participant.

Participant no.	Group	Height (m)	Body Mass (kg)	BMI (kg/m^2)	Baseline Cooper run (km)	Week 4 Cooper run (km)	Post Cooper run (km)	ΔCooper run pre vs post (%)	Total training load (A.U.)
1	EG	1.83	74.0	22.1	2.45	2.17	2.26	-7.76	43860.0
2	EG	1.85	90.3	26.4	2.07	2.44	2.54	22.71	46510.0
3	EG	1.68	59.0	21.0	1.76	1.98	2.09	18.75	43860.0
4	EG	2.04	108.0	26.0	2.33	2.61	2.63	12.88	47705.0
5	EG	1.80	89.0	27.5	2.17	2.30	2.41	11.11	52985.0
6	EG	1.65	166.0	61.3	1.61	1.88	1.93	20.00	43860.0
7	EG	1.75	69.0	22.5	2.54	2.67	2.71	6.69	46897.5
8	EG	1.85	105.0	30.7	2.24	1.96	2.09	-6.47	43860.0
9	EG	1.78	89.0	28.1	1.40	1.64	1.71	22.14	43860.0
10	EG	1.80	84.8	26.2	2.31	2.43	2.42	4.76	43860.0
11	EG	1.80	89.0	27.5	2.43	2.51	2.59	6.62	66215.1
12	EG	1.75	61.2	20.0	1.55	1.61	1.63	5.16	43860.0
13	EG	1.73	57.0	19.0	3.42	3.59	3.64	6.43	64267.5
14	EG	1.79	97.5	30.6	2.66	2.43	2.97	11.65	43860.0
15	EG	1.80	76.5	23.6	1.88	2.21	2.28	21.28	43860.0
16	EG	1.69	60.7	21.3	1.85	2.20	2.24	20.87	43860.0
17	EG	1.88	91.2	25.8	2.41	2.57	2.64	9.33	53660.0
18	EG	1.81	107.5	32.8	2.20	2.35	2.39	8.64	43860.0
19	EG	1.73	74.9	25.0	2.88	3.01	3.15	9.50	73435.0
20	EG	1.70	75.0	25.9	1.80	2.12	2.29	26.79	43860.0
21	EG	1.84	73.0	21.5	2.59	2.82	2.86	10.56	43860.0
22	CG	1.65	63.0	23.1	1.95	1.98	1.93	-0.83	5250.0
23	CG	1.73	75.0	25.1	2.25	2.32	2.17	-3.56	8150.0
24	CG	1.68	75.0	26.6	1.63	1.43	1.60	-1.84	5400.0
25	CG	1.65	54.0	19.8	1.69	1.72	1.61	-4.76	14655.0
26	CG	1.63	54.0	20.4	2.05	1.99	2.05	0.00	24360.0
26	CG	1.70	80.4	27.8	1.87	1.85	1.89	1.07	3829.5
28	CG	1.87	71.0	20.3	1.98	2.29	2.43	22.76	9300.0
29	CG	1.65	88.2	32.4	1.91	2.02	2.04	6.81	18820.0
30	CG	1.65	61.6	22.6	1.30	1.30	1.38	6.15	19850.0
31	CG	1.64	54.0	20.1	2.22	2.30	2.20	-0.90	30588.0
32	CG	1.73	101.3	33.8	2.30	2.24	2.27	-1.30	44656.0
33	CG	1.85	69.0	20.2	2.65	2.94	2.94	10.94	36000.0
34	CG	1.63	51.0	19.3	2.54	2.61	2.65	4.33	30480.0
35	CG	1.85	88.0	25.7	2.70	2.75	2.82	4.17	104085.0
36	CG	1.73	75.3	25.2	2.63	2.41	2.44	-7.22	7014.4
37	CG	1.67	68.0	24.4	1.90	1.98	1.87	-1.69	13900.0
38	CG	1.82	105.0	31.7	2.42	2.51	2.05	-14.74	18280.0
39	CG	1.77	76.0	24.3	1.51	1.55	1.48	-1.94	12210.0
40	CG	1.88	98.7	27.9	2.00	2.00	2.09	4.83	19200.0
41	CG	1.62	68.3	26.0	1.90	1.92	1.96	3.13	21880.0
42	CG	1.66	63.0	22.9	2.10	2.37	2.21	4.64	16600.0
43	CG	1.66	62.5	22.6	2.28	2.14	2.31	1.40	23675.0
44	CG	1.75	74.8	24.4	1.67	1.68	1.74	4.17	18130.0
45	CG	1.76	82.5	26.6	1.88	2.06	1.92	1.56	36882.7
