# Supplementary material for: Responsiveness to endurance training can be partly explained by the number of favorable single nucleotide polymorphisms an individual possesses
Source: PLoS One. 2023 Jul 20;18(7):e0288996. doi: 10.1371/journal.pone.0288996 (PMC10358902; doi:10.1371/journal.pone.0288996)
Supplement: Attachment — Submitted filename: Response to Reviewers.docx [file pone.0288996.s004.docx]

**Rebuttal letter 'Response to Reviewers' comments**

Firstly, we would like to thank the editor and reviewers for taking the time to review our manuscript entitled, Responsiveness to endurance training can be partly explained by the number of favorable single nucleotide polymorphisms an individual possesses for PLOS ONE [PONE-D-22-31088] - [EMID:ec37305078e9f797].

We are pleased that the decision of the manuscript has minor revisions, we therefore accept the invitation to submit a revised version of the manuscript. Attached in this letter is a table of the points highlighted with our responses in red.

With this we have also attached a revised manuscript with the track changes and also a version where the track changes are not highlighted but included.

We hope that these revisions made are satisfactory, and we look forward to hearing back from you. Please do not hesitate to contact us if you require further information.

Thank you again.

Kind regards,

Dr Henry Chung (Corresponding author)

| Academic editor and reviewer(s) points | Response to comments |
| --- | --- |
| Reviewer 1: | Authors: |
| 1. Please ensure that your manuscript meets PLOS ONE's style requirements, including those for file naming. The PLOS ONE style templates can be found at  [https://journals.plos.org/plosone/s/file?id=wjVg/PLOSOne_formatting_sample_main_body.pdf](https://linkprotect.cudasvc.com/url?a=https%3a%2f%2fjournals.plos.org%2fplosone%2fs%2ffile%3fid%3dwjVg%2fPLOSOne_formatting_sample_main_body.pdf&c=E,1,GOQoNvuPMbOb8tHSK8lTur5SFq1GQL689bHc1bVqul_HiyllnPTFsCLcuRxnZK1Y33fPLNiOz7VUZBe0JdK2xpnNkYSsq7WwDC-02abL-x_RlgZdSgbppe8b&typo=1&ancr_add=1) and  [https://journals.plos.org/plosone/s/file?id=ba62/PLOSOne_formatting_sample_title_authors_affiliations.pdf](https://linkprotect.cudasvc.com/url?a=https%3a%2f%2fjournals.plos.org%2fplosone%2fs%2ffile%3fid%3dba62%2fPLOSOne_formatting_sample_title_authors_affiliations.pdf&c=E,1,ha5eryJn8_EFKhbjb5FUfTAxccVt0-RaYFVBHetheeuEbOyMwr4vfm_GFVOgT2nRSbLGinefzjvURAASjiELZHxWIw-6ant2iSEiL0slcWMFVZ2kyao,&typo=1&ancr_add=1) | In respect to both documents the formatting for   1. - Line 351, ‘Fig.4.’ changed to ‘Fig 4’.  - Equations in ‘Training intervention” have been changed using equation tool in word in line 168-173. - Table 1 has been edited on page 14. Instead of using * we have now used ^a^ and ^b^ as directed on the sample. - All captions have been changed from font 9 to font 11 to match the sample document.  2. The title, author and affiliations are aligned with the guidelines. We have changed the contribution section in page 1 now to reflect similarly to the sample that all authors contributed equally to the work. |
| 2. Thank you for stating the following financial disclosure:  The funders had no role in study design, data collection and analysis, decision to publish, or preparation of the manuscript.  At this time, please address the following queries:  a) Please clarify the sources of funding (financial or material support) for your study. List the grants or organizations that supported your study, including funding received from your institution.  b) State what role the funders took in the study. If the funders had no role in your study, please state: “The funders had no role in study design, data collection and analysis, decision to publish, or preparation of the manuscript.” c) If any authors received a salary from any of your funders, please state which authors and which funders. d) If you did not receive any funding for this study, please state: “The authors received no specific funding for this work.”  Please include your amended statements within your cover letter; we will change the online submission form on your behalf. | In respect to these questions there might have been some confusion. This study did not have any funders. The statement for the application process might need to be changed in this respect.   a) There was no source of funding. The manuscript acknowledgement section also disclosed the funding formation as “research did not receive any specific grant from funding agencies in the public, commercial, or for-profit sectors”. b) There were no funders for this project, we consulted a commercial genetic company to do the genotypes, where we paid for the genotypes on behalf of the participants. The genotype company had no role within this project and did the genotype as they would for any public buyer of the product. c) No authors received any salary or benefits from using the genetic company. d) The authors received no specific funding for this work. This was already included in the acknowledgement section but now has been included in the cover letter. Thank you for clarifying these steps and also willing to change the online submission on our behalf.   We hope these small changed and responses clarify the situation and are satisfactory. |
| 3. Please include captions for your Supporting Information files at the end of your manuscript, and update any in-text citations to match accordingly. Please see our Supporting Information guidelines for more information: [http://journals.plos.org/plosone/s/supporting-information.](https://linkprotect.cudasvc.com/url?a=http%3a%2f%2fjournals.plos.org%2fplosone%2fs%2fsupporting-information.%26nbsp%3b&c=E,1,YR2VAEwMQtXquhNhBGaaSQXuZCv48RC4PqrlrM6AcyyQijDaOMSvqNd3mB-LkmrbfG3O3uVufOYNEfg-6h-xcKUG_l9eBYoLmehfJih3fkLJ&typo=1&ancr_add=1) | - We have now included both the supporting documents / files captions at the end of the manuscript in ‘Supporting information’ section. - Additionally we have included S1 File and S2 File in-text in the manuscript. - Both the supporting files have also been renamed and re-uploaded with the correct file names now. Thank you for pointing this out for us. |
| 4. Please review your reference list to ensure that it is complete and correct. If you have cited papers that have been retracted, please include the rationale for doing so in the manuscript text, or remove these references and replace them with relevant current references. Any changes to the reference list should be mentioned in the rebuttal letter that accompanies your revised manuscript. If you need to cite a retracted article, indicate the article’s retracted status in the References list and also include a citation and full reference for the retraction notice. | Thank you for pointing this out.  The entire reference section has been updated and checked. We are now confident it is correct. All intext citations have also been updated to match the reference list. |
